# Protein structural class prediction based on an improved statistical strategy

**DOI:** 10.1186/1471-2105-9-S6-S5

**Published:** 2008-05-28

**Authors:** Fei Gu, Hang Chen, Jun Ni

**Affiliations:** 1 Department of Biotechnology, College of Life Sciences, Zhejiang University, Hangzhou, 310027, China; 2 Department of Biomedical Engineering, College of Biomedical Engineering and Instrument Science, Zhejiang University, Hangzhou, 310027, China; 3 Department of Radiology, College of Medicine, The University of Iowa, Iowa City, IA 52242, USA

## Abstract

**Background:**

A protein structural class (PSC) belongs to the most basic but important classification in protein structures. The prediction technique of protein structural class has been developing for decades. Two popular indices are the amino-acid-frequency (AAF) based, and amino-acid-arrangement (AAA) with long-term correlation (LTC) – based indices. They were proposed in many works. Both indices have its pros and cons. For example, the AAF index focuses on a statistical analysis, while the AAA-LTC emphasizes the long-term, biological significance. Unfortunately, the datasets used in previous work were not very reliable for a small number of sequences with a high-sequence similarity.

**Results:**

By modifying a statistical strategy, we proposed a new index method that combines probability and information theory together with a long-term correlation. We also proposed a numerically and biologically reliable dataset included more than 5700 sequences with a low sequence similarity. The results showed that the proposed approach has its high accuracy. Comparing with amino acid composition (AAC) index using a distance method, the accuracy of our approach has a 16–20% improvement for re-substitution test and about 6–11% improvement for cross-validation test. The values were about 23% and 15% for the component coupled method (CCM).

**Conclusion:**

A new index method, combining probability and information theory together with a long-term correlation was proposed in this paper. The statistical method was improved significantly based on our new index. The cross validation test was conducted, and the result show the proposed method has a great improvement.

## Background

Protein function is strongly related to its structure. Analysis of protein functions becomes a fundamental research domain to comprehend its structures. Nowadays, with the increased number of parsed structure entries in bioinformatics databases, it is important to do classification of protein structures in bioinformatics research. Scientists had developed various methodologies for the classification of protein structures. For example, based on the structure types and the arrangements of secondary structural elements, Levitt and Chothia [1] proposed a method to recognize ten protein classes, four principal and six small classes of a protein structure. Biological scientists common focus on the first four principal classes. They are all-*α*, all-*β*, *α*/*β*, and *α*+*β *classes, respectively. Therefore, the prediction of the four principal protein structural classes is the foundation in the field of protein analysis. In the fundamental study, many indices and methods were proposed to predict protein structural class [2-7]. The commonly-used indices and their corresponding methods are described briefly in the following.

Nishkawa [8] et al. found that protein structural classes are related to their amino acid compositions (AAC). Based on this hypnosis, Chou [9,10] proposed standard vectors from amino acid composition in proteins. The statistics-based indices are 20-dimensional vectors, through which each variant corresponds to one amino acid occurrence frequency in protein sequence. Although these indices can be considered the eigenvector of a sequence, the information is insufficient enough to reflect the correlation among residues. Another weakness is that the statistics indices can not reflect the biological significance commendably. Accordingly, several methods were proposed such as the distance-based algorithm [11,12], component-coupled-based algorithm [13-15], support vector machine (SVM) based algorithm [16] and others [17,18].

Alternatively, people can introduce protein-structural-class prediction index, which is based on the residues' arrangement and correlation in analysis of proteins. Such index method that uses various physiochemical properties has been experimented and adopted in the prediction. For example, Bu et al. [19] found that the auto-correlation function (ACF) of average non-bonded energy can represent the protein structural class with a better accuracy of prediction. Although a long-term correlation between different residues was considered, it did not include the statistical characteristics of sequences.

In this paper, a new index method is proposed. The method is based on the information and probability theories. In this method, a residue occurrence frequency is used instead of physiochemical indices for long-term correlation calculation. The statistical strategy of residue occurrence frequency is changed from a single sequence to a whole-training dataset. The results showed that the accuracy is significantly improved.

## Methods

Suppose the whole dataset *S *contains *N *sequences, and this dataset can be divided into *m *(in this paper, we set *m *= 4; without losing generality) subsets *S*_*ξ *_(*ξ *= 1, 2,......, *m*), thus,

(1) *S *= *S*_1 _∪ *S*_2 _∪ ...... ∪ *S*_*m *_(*m *= 4)

The number of sequences in each subset is given by *n*_*ξ*_; thus the total number of sequence, $N=\sum_{\xi =1}^{m}n_{\xi }$

Chou et al. [9] proposed an index based on the amino acid composition (AAC) frequency in a sequence (Equation 2–4), i.e,

(2) $$\begin{array}{l} X_{k}^{\xi }={\left[x_{k,1}^{\xi },x_{k,2}^{\xi }......x_{k,20}^{\xi }\right]}^{T}\\ \left(k=1,2,...,n_{\xi };\xi =1,2,...,m\right) \end{array}$$

Where $x_{k,1}^{\xi },x_{k,2}^{\xi }......x_{k,20}^{\xi }$ are the normalized occurrence frequencies of 20 residues for the *k*^th ^protein $X_{k}^{\xi }$ in the subset *S*_*ξ*_, and *T *stands for the transpose symbol.

The average occurrence frequencies or the so-called standard vector for subset *S*_*ξ *_is represented by

(3) $${\bar{X}}^{\xi }={\left[{\bar{x}}_{1}^{\xi },{\bar{x}}_{2}^{\xi },......,{\bar{x}}_{20}^{\xi }\right]}^{T},\left(\xi =1,2,...,m\right)$$

where

(4) $${\bar{x}}_{i}^{\xi }=\frac{1}{n_{\xi }}\sum_{k=1}^{n_{\xi }}x_{k,i}^{\xi },\left(i=1,2,...,20\right)$$

Since Chou's great contribution, many methods that are based on residue composition were proposed. The *n*-order component coupled method was one of them. When *n *= 0, this algorithm degenerated to the amino acid composition (AAC) method. In the case when *n *= 1, the corresponding indices can be expressed in terms of a 20 × 20 conditional probability matrix [20]. And if *n *> 1, the *n*-order component coupled components can be expressed in terms of a multi-dimensional conditional probability matrix. In those residue-composition-based methods, the size of statistics samples must be largely enough. However, the present statistical approach requires to calculate the probability of 20 amino acids or the conditional probability for one sequence. In this way, the conditional probabilities, especially the high-order coupled components, can not be calculated accurately since the length of each protein sequence is not long enough. For any *n *= 0 coupled component, the influence of amino acid that nearby was not considered. With the increase of *n*, the long-term interaction between the residues at different positions in a same sequence can be reflected; which it is of computational complexity.

In order to solve these problems mentioned above, a new method with an innovative index is proposed in this paper, which can be summarized as follows:

First, a new statistical approach was proposed. The amino acids' component frequency of each entire class (expressed in Equation 5, rather than Equation 4) is calculated, instead of the occurrence frequencies of different residues for a certain protein in each class.

(5) $${\bar{x}}_{i}^{\xi }=\frac{\sum_{k=1}^{n_{\xi }}x_{k,i}^{\xi }\times l_{k}^{\xi }}{\sum_{k=1}^{n_{\xi }}l_{k}^{\xi }},\left(i=1,2,......,20\right)$$

where $l_{k}^{\xi }$ is the size of *k*^th ^sequence length for the subset *S*_*ξ*_, and the other parameters remain the same definitions as in Equation 4.

Secondly, we develop a method to improve the component coupled algorithm. Conditional probabilities of different amino acids that have different correlation lengths can be calculated. To simplify the calculation procedure, only a 2-dimensional (20 × 20) matrix is introduced to express any possible distances between residues. The conditional probability can be expressed as *P*_*d *_(*a*_*i*_/*a*_*j*_), where the subscript *d *is the distance between the residue *a*_*i *_and *a*_*j*_, that is, *d *= *i*-*j*. For each *d*, one has

(6) $$\begin{array}{cc} \sum_{j=1}^{20}\sum_{i=1}^{20}P_{d}(a_{i}/a_{j})=1, & \left(d=0,1,2,......\right) \end{array}$$

According to the theory of the probability multiplication:

(7) *P*_*d *_(*a*_*i*_/*a*_*j*_) = *P*_*d*_(*a*_*i*_, *a*_*j*_)/*P*_*d*_(*a*_*j*_)

In Equation 7, *P*_*d *_(*a*_*i*_, *a*_*j*_) and *P*_*d*_(*a*_*j*_) can be easily computed from protein sequences, and the conditional probability *P*_*d*_(*a*_*i*_/*a*_*j*_) can also be calculated.

For the case that *d *+ *j *exceeds the length of the sequence, the cyclic boundary condition can be used. The residue at which its position is equal to the remainder of *d *+ *j *and the length of sequence can be considered.

The third step is to determine the indexation of the conditional probability matrix for prediction. The information content of conditional probability is used as the quantification index. For each residue (*a*_*j*_) in an undetermined sequence, the index of the *d*-interval can be calculated as:

(8) *I*_*d*_(*a*_*j*_) = -log *P*_*d*_(*a*_*i*_/*a*_*j*_), (*j *= 1, 2,......*l*)

In this natural logarithm expression, *l *is the length of sequence *k*. For all the residues in the sequence *k*, the total information content can be obtained by

(9) *I*_*d *_= *I*_*d *_(*a*_1_) + *I*_*d *_(*a*_2_) + ...... + *I*_*d *_(*a*_*l*_)

To consider multi-residue effects on some amino acids, the information contents with different distances can be accumulated to form the whole information contents, *I*_*w*_, i.e.,

(10) *I*_*w *_= *I*_*a *_+ *I*_*a*+1 _+ ... + *I*_*b *_(*a*, *b *= 0, 1,...*l*, *b *≥ *a*)

From Equation 8, we can find that the larger the conditional probability is, the smaller the information content is. Hence, the prediction result with minimum total information content should be considered in a predicted class in our method.

(11) $$I_{d}=\min(I_{d}^{1},I_{d}^{2},I_{d}^{3},I_{d}^{4})$$

(12) $$PD(\xi )=\left\{ \begin{array}{l} \alpha (I_{d}=I_{d}^{1})\\ \beta (I_{d}=I_{d}^{2})\\ \alpha +\beta (I_{d}=I_{d}^{3})\\ \alpha /\beta (I_{d}=I_{d}^{4}) \end{array}\right.$$

where *PD *is the predicted result.

## Dataset and results

### Dataset

In order to comprehensively perform our statistical studies, the latest version (version 1.71 updated on 24 January 2007) of the database SCOP [21] was used. Four classes' sequences – including 1267 in *α *class, 1424 in *β *class, 1682 in *α*/*β *class and 1551 in *α*+*β *class – with the similarity less than 30% were selected (the reason why using this dataset will be explained in discussion part in detail). After removing the uncertainty sequences that contain the letter *x *in sequence, the total numbers are 1250, 1375, 1565 and 1524, respectively (see additional files 1 and 2). According to the cross-validation principle, a whole sequence was divided into two subsets, randomly. The training and prediction sets were non-homologous and we selected a number that is large enough for training and test (about 20 times more than the size of dataset used in [9]).

### Results

To test the feasibility, verification, and applicability of our index and method, the cross-validation [22] method was used in our study. The total sequences including 4 classes were randomly divided into 2 datasets, i.e., the training and the prediction datasets. The training dataset contains 2856 sequences, and the prediction dataset contains 2858 sequences.

Two traditional indices, AAC and ACF mentioned above, were used to compare with the results from our method. Three methods, mainly, the Hamming distance algorithm (DH), the Euclidean distance algorithm (DE) and the component coupled algorithm (CC), were used to assess the indices.

For the AAC index, the results of DH, DE and CC method were shown in Table 1 and 2.

**Table 1:** Training dataset using AAC index

Method	*α *class	*β *class	*α*/*β *class	*α*+*β *class	Overall
DH(%)	61.44	59.39	46.42	25.46	47.23
DE(%)	65.12	60.99	49.23	26.38	49.44
CC(%)	91.68	68.12	45.52	23.10	55.07

**Table 2:** Prediction dataset using AAC index

Method	*α *class	*β *class	*α*/*β *class	*α*+*β *class	Overall
DH(%)	61.76	60.32	46.36	25.33	47.48
DE(%)	65.76	61.19	48.91	27.17	49.76
CC(%)	89.92	64.97	42.71	19.29	52.13

For the auto-correlation based method, we found that our method with hydrophobicity based indices has a higher accuracy value than the one with other physiochemical properties. We used the Kyte and Doolittle [23] hydrophobicity values respectively, and the number of the auto-correlation function length is listed in Table 3 and 4.

**Table 3:** Training dataset using Kyte and Doolittle ACF index

Method	*α *class	*β *class	*α*/*β *class	*α*+*β *class	Overall
DH(%)	57.60	65.50	41.18	23.10	45.80
DE(%)	61.28	69.14	46.04	24.15	49.09

**Table 4:** Prediction dataset using Kyte and Doolittle ACF index

Method	*α *class	*β *class	*α*/*β *class	*α*+*β *class	Overall
DH(%)	59.20	62.35	38.06	23.88	44.75
DE(%)	61.92	68.60	42.78	22.57	47.80

In our experiments, different numbers of long-term correlations were tested, and the distance (*d*) between 2 and 4 shows to have a better result of accuracy. The results for training dataset and prediction dataset were shown in Table 5.

**Table 5:** Training and prediction dataset using our index*

Dataset	*α *class	*β *class	*α*/*β *class	*α*+*β *class	Overall
Training (%)	78.24	71.18	63.81	49.87	65.02
Prediction (%)	70.08	63.23	57.34	34.51	55.46

*The results were obtained by considering the long-term correlations with the distance 2–4.

**Figure 1:**
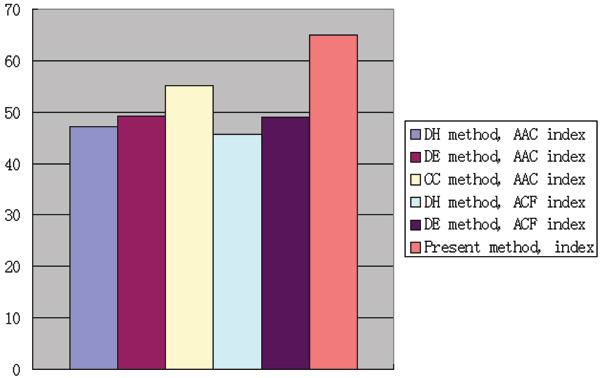
Accuracy of 3 indices for the training dataset

**Figure 2:**
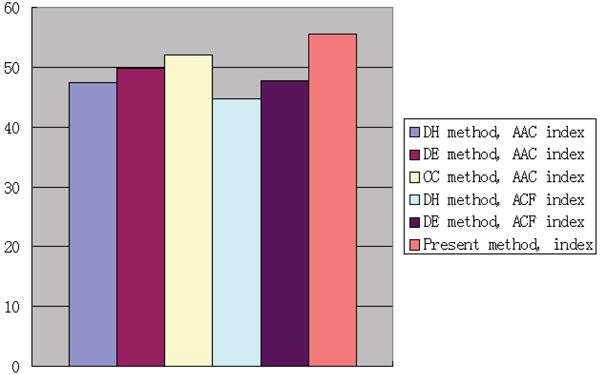
Accuracy of 3 indices for the prediction dataset

The comparison of training and prediction results calculated by three different indices was illustratively presented in Figure 1 and 2. We found that our index has the best accuracy in protein structural class prediction. With the same index, the method DE always obtained better accuracy than the method DH.

## Discussion

We will discuss the dataset, since it is the most important part in evaluating different indices and methods. People usually use the frequently-used dataset which includes several hundred sequences [10]. It is not relatively reliable enough, relevant to a given dataset scale. Another critical issue is the high sequence similarity. Let's take the 277 dataset [10] as an example. The 277 contains 277 protein domains extracted from the SCOP database.

The remarkable pair-wise similarity can be found in each class after multiple sequence alignment is conducted. For instance, in an alpha class, we found that there are several groups of identical sequences; the biggest one contains about 20 sequences (see additional files 1 and 2). After we conducted pair-wise alignment among these 20 sequences, we found that the sequence similarity was over 85%; indicating that these sequences are very identical to each other. The finding happens when we used other 3 classes. Such a high sequence similarity existed in the both training and test datasets; certainly violating the principle of cross validation. Therefore, we suspended such dataset for a reliable result.

In order to clearly emphasize the importance of selected dataset, we compared the three above methods from two different datasets. The amino acid composition index was used in this comparison study. The re-substitution and cross validation tests were designed and implemented for feature evaluations.

**Figure 3:**
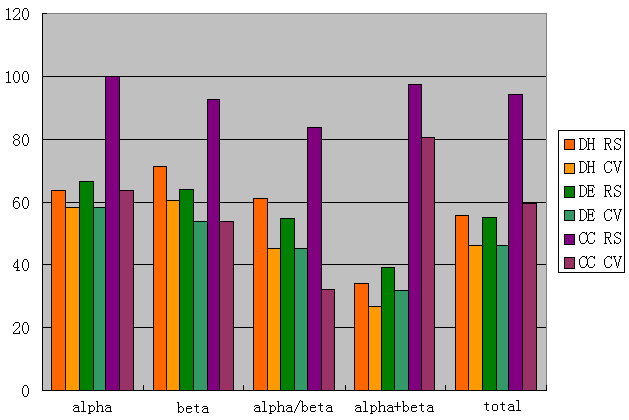
Accuracy of amino acid composition index using the 138 dataset.  DH, DE and CC mean the Hamming distance method, the Euclidean distance method and the component coupled method respectively.  RS means the re-substitution text, and CV corresponds to the cross validation text.

For the dataset including 138 sequences [10], the accuracy for re-substitution test and jack-knife test are shown in Table 6 and 7, and plotted in Figure 3, respectively.

**Table 6:** The 138 dataset with re-substitution test^1^

Class	Alpha	Beta	Alpha/beta	Alph+beta	total
DH hit number	23	20	19	14	76
DE hit number	24	18	17	16	75
CC hit number	36	26	26	40	128
Class number	36	28	31	41	136
DH accuracy(%)	63.89	71.43	61.29	34.15	55.88
DE accuracy(%)	66.67	64.29	54.84	39.02	55.15
CC^2 ^accuracy(%)	100	92.86	83.87	97.56	94.12

^1^note that the total number was a little bit different from reference 10, since the PDB database was updated in recent years.

^2^CC means the component coupled method.

**Table 7:** The 138 dataset with jack-knife test.

Class	Alpha	Beta	Alpha/beta	Alph+beta	total
DH hit number	21	17	14	11	63
DE hit number	21	15	14	13	63
CC hit number	23	15	10	33	81
Class number	36	28	31	41	136
DH accuracy (%)	58.33	60.71	45.16	26.83	46.32
DE accuracy (%)	58.33	53.57	45.16	31.71	46.32
CC accuracy (%)	63.89	53.57	32.26	80.49	59.56

**Figure 4:**
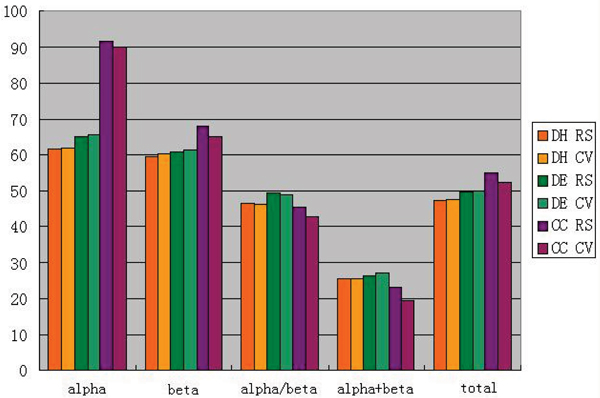
The accuracy of amino acid composition index using the 5714 dataset.  DH, DE and CC mean the Hamming distance method, the Euclidean distance method and the component coupled method, respectively.  RS means the resubstitution text, and CV corresponds to the cross validation set.

Our dataset is summarized in Table 8 and 9 with a total number of 5714 sequences, 2856 for training dataset and 2856 for testing dataset (see Figure 4).

**Table 8:** The 2856 dataset with re-substitution test

Class	Alpha	Beta	Alpha/beta	Alph+beta	total
DH hit number	384	408	363	194	1349
DE hit number	407	419	385	201	1412
CC hit number	573	468	356	176	1573
Class number	625	687	782	762	2856
DH accuracy(%)	61.44	59.39	46.42	25.46	47.23
DE accuracy(%)	65.12	60.99	49.23	26.38	49.44
CC accuracy(%)	91.68	68.12	45.52	23.10	55.07

**Table 9:** the 2858 dataset with cross validation test*

Class	Alpha	Beta	Alpha/beta	Alph+beta	total
DH hit number	386	415	363	193	1357
DE hit number	411	421	383	207	1422
CC hit number	562	447	334	147	1490
Class number	625	688	783	762	2858
DH accuracy(%)	61.76	60.32	46.36	25.33	47.48
DE accuracy(%)	65.76	61.19	48.91	27.17	49.76
CC accuracy(%)	89.92	64.97	42.71	19.29	52.13

*This 2858 dataset is totally different from table 6. The two datasets were obtained from the random separation of the 5714 dataset. We used the dataset in table 6 as training samples, and the sequences in table 7 were test samples. This cross validation method can be considered more reliable than jack-knife test.

From Table 6 and 7, one can find that the prediction accuracy is very high for all three methods. This is because that the 138 dataset, just like the 277 dataset, is homologous, which means some sequences are almost the same. We can also find an interesting phenomenon that the accuracy of DH and DE are relatively higher in a cross validation test than that is in re-substitution test. It is mainly because these methods are insensitive to dataset, which means that there is a good extrapolating property in these algorithms. Comparing with CC and SVM, the total accuracy of our method is much better. However, like many advanced methods, the accuracies of re-substitution and cross validation tests are significantly different.

Traditional methods are usually based on simple criterions, while new-developed algorithms have more complicated rules. More prior probability information made current methods more accurate. However, this information must strongly rely on dataset. Fortunately, with an increased number of parsed-sequences, scientists can solve this problem commendably.

Generally speaking, using three above methods, the accuracy of dataset 5714 is much lower than one of the dataset 138. The 138 dataset is unreliable due to its high sequence similarity. However, in cross-validation test, the accuracy of DH and DE in 5714 dataset is much higher than that in 138 dataset. This illuminates that with an increase of dataset scale, one can improve the extrapolation of algorithms remarkablely.

From Table 1, 2, 3 and 4, we found that the accuracy is obviously decreased, compared with the result mentioned before. This is mainly because that the dataset we used are now larger and much different from the one used before. Therefore, the traditional methods had to be improved with an increase of sample size.

Table 1, 2, 3 and 4 also tell us that the difference of accuracy between the training and the prediction datasets is quite small. Therefore, the generalization of these methods is pretty good. It is because there are very few restriction conditions and technical manipulations in traditional methods that avoid a fluctuation between the training and test results by some techniques.

Using our method, the accuracy is between 6% and 16% higher than in the traditional methods. This is because long-term concepts are introduced and the conditional probability is used instead of physiochemical indices; thus to avoid the errors influenced by other parameters. In our test, distance (*d*) value is between 2 and 4, the accuracy is high. This phenomenon is a good accordance with the frequency characteristics of proteins. As we all know, most alpha helices are 3.6 residues per cycle, which means that a hydrogen bond bridges current residue and the residue 3 or 4 positions behind. Most beta strands have 2 residues per strand cycle, which reflects a strong interaction between two residues in a 2-position interval.

The advantage of our method can be concluded into three aspects:

• In our method, the long-term correlation factor is considered without any other physiochemical parameters.

• The accuracy is significantly improved for about 6–16% comparing with two traditional indices.

• The merits in both two traditional methods are inherited. That is, the residue composition frequency and the amino acid arrangement.

However, there still exit some problems, which motivate our future study.

• In our method, we must calculate the correlation between *d *residues. For the situation that the residue position is near the end of a sequence, the residue *d *sites behind may exceed the length of the sequence. In such case, the boundary process is crucial to the final result. For convenience, the cyclic boundary condition is used hereby. However, such approach is not biologically significant, and it is not quite reliable. To solve this problem, we are planning to test different types of extended boundary conditions.

• The presented method only calculate the correlation between certain residue and the residue *d *positions behind. This is a "one-side" statistical work, and the information can not be extracted enough. The calculation of the correlation between the target residue and the residues different sites before and after is necessary to solve the problem.

## Conclusion

In this paper, a new method by new indices is proposed. A reliable dataset with large number of entries and low sequence similarity is used to train and test our algorithm. The result showed that our method has a higher accuracy than the ones in traditional methods. The application of conditional probability and information content shows that the protein structural prediction can be largely improved by combining the information theory with the probability theory.

## Competing interests

The authors declare that they have no competing interests.

## Supplementary Material

Additional file 1 **Datasets**. The 5172 dataset including 1250 alpha class sequences (alphatotal sheet), 1375 beta class sequences (betatotal sheet), 1565 alpha/beta class sequences (aabtotal sheet) and 1524 alpha+beta class sequences (apbtotal sheet). The training dataset including 625 alpha class sequences (alphatrain sheet), 687 beta class sequences (betatrain sheet), 782 alpha/beta class sequences (aabtrain sheet) and 762 alpha+beta class sequences (apbtrain sheet). The training dataset including 625 alpha class sequences (alphapre sheet), 688 beta class sequences (betapre sheet), 783 alpha/beta class sequences (aabpre sheet) and 762 alpha+beta class sequences (apbpre sheet). Click here for file

Additional file 2 **Sequences and sequence alignment result**. Traditional datasets were always included the sequences with high sequence similarity. Take the 277 dataset as an example, the biggest identical group for alpha class was shown below: This group contains 20 sequences as in file 'additional file 2.txt'. Then the pair-wise sequence alignment was performed using the program FASTA, version 3.3, the result was also represented in file 'additional file 2.txt'. From this file, we can find a extremely high sequence similarity among these sequences. This situation made the dataset unreliable. Click here for file
